# When *pull*ulanase needs a little push: MyoAAV capsids enhance gene therapy for GSD IIIa

**DOI:** 10.1016/j.omtm.2025.101648

**Published:** 2025-11-29

**Authors:** Sree Venigalla, Christina A. Pacak

**Affiliations:** 1 Greg Marzolf Jr. Muscular Dystrophy Center and Department of Neurology, University of Minnesota Medical School, Minneapolis, MN, USA; 2 Center for Genome Engineering, University of Minnesota, Minneapolis, MN, USA; 3 Stem Cell Institute, University of Minnesota, Minneapolis, MN, USA

## Main text

Numerous adeno-associated virus (AAV) based gene therapies are currently being evaluated in human clinical trials and six have been approved by the United States Food and Drug Administration (US-FDA). AAV9 in particular, has been highly successful as the AAV capsid used in onasemnogene abeparvovec (Zolgensma), a gene therapy to treat infants with spinal muscular atrophy (SMA). While overall this therapy is tremendously effective, elevated liver function tests are a common adverse event in the treated population, and careful monitoring and corticosteroid administration are needed to mitigate risks.^1^ These patient safety concerns are even more prevalent in muscle-specific disorders where the extremely high doses of AAV9 needed for therapeutic effect have caused serious adverse events in clinical trials. One promising strategy to improve AAV efficacy in specific tissues (and avoid others) involves engineering AAV capsids through targeted modification or directed evolution to create variants with enhanced delivery properties that can more precisely transduce specific cell types. In a recent issue of molecular therapy methods and clinical development, Liao et al. compared the efficacy of AAV9 to two capsids from the MyoAAV series in their ability to deliver the bacterial pullulanase transgene for the treatment of glycogen storage disease type IIIa (GSD IIIa).^2^ The investigators found that the MyoAAV4A-based vector (at a 10-fold lower dose) achieved significantly better therapeutic outcomes in skeletal muscles and heart than an AAV9-based vector while also successfully treating the liver. These results provide strong rationale for incorporation of myotropic vectors in human clinical trials to reduce effective vector dosages.

GSD IIIa is a rare recessive disorder caused by pathogenic variants in the gene *AGL*, which encodes the glycogen debranching enzyme (GDE). GDE is a bifunctional enzyme responsible for glycogen degradation via glucotransferase and amylo-glucosidase activities. GSD IIIa is a challenging disorder for therapeutic development due to its multi-system involvement caused by the progressive accumulation of abnormal, partially degraded glycogen (also called limit dextrin) in hepatocytes and myocytes. To date, treatment for GSD IIIa includes dietary interventions to manage hypoglycemia, but progressive cardiomyopathy and skeletal myopathy persist, severely impacting quality of life and often leading to premature death. With no FDA approved treatments available, gene therapy is an attractive option for GSD IIIa therapeutic development but an approach with widespread and durable transduction across the heart and skeletal muscles with a low effective viral dose is crucial for success.

The MyoAAV4a and MyoAAV4e vectors evaluated in this study are both variants from a newly developed class of AAVs (the AAVMYO and MyoAAV series) that have been specifically engineered to target skeletal and cardiac muscle and often to also avoid the liver.^3^ As GSD IIIa affects multiple tissues, including the heart, skeletal muscles, and liver, it is somewhat unique among muscle disorders in that the liver does not need to be entirely avoided and if found to be safe and effective, liver treatment is desirable. The AAVMYO series variants are based upon AAV9 or chimeras from shuffled libraries of multiple capsids including AAV9^4^ while the MyoAAV series variants were developed through directed evolution of AAV9.^5^ A feature that is in common across these myotropic capsids is the arginine, glycine, aspartic acid motif (commonly referred to as the “RGD” motif) that enables capsid interaction with integrin receptors and is the key that facilitates enhanced tropism for skeletal muscle and heart and reduced (but not entirely eliminated) affinity for the liver.

Incorporating the human *AGL* cDNA into an AAV-based gene delivery vector is not trivial due to its large size and the packaging constraints of AAV. The investigative group had previously overcome this size limitation through the use of an AAV vector that expressed a smaller bacterial version of GDE, pullulanase (*Pull*) that easily fits within AAV vectors.^6^ The group administered a combination of AAVs to GSDIIIa mice; one containing an immunotolerizing liver-specific promoter (LSP) and the other a ubiquitous CMV enhancer/chicken β-actin (CB) promoter to drive *Pull* expression.^6^ They further refined their approach through use of an elegantly designed, dual-promoter gene cassette that expresses *Pull* under control of both the LSP and the CB promoters.^7^

In this most recent iteration of the therapy, the investigators sought to improve efficacy and reduce the effective dose to address known AAV9 safety concerns associated with liver toxicity, systemic immune response mediated inflammation, and dose-dependent complement activation that have been previously observed.^8^ Liao et al. packaged their previously validated dual-*Pull* expression cassette into MyoAAV4A, MyoAAV4E, and AAV9 vectors and administered a single intravenous dose of 1×10^13^ vg/kg to 12-week-old male GSD IIIa mice. Evaluations of vg content, transgene expression, pullalanase activity (in heart, skeletal muscles, and liver), glycogen storage content, muscle function tests, and liver enzyme levels were performed.^2^ It was clear that while both MyoAAV4A-dual-*Pull* and MyoAAV4E-dual-*Pull* were significantly better than AAV9-dual-*Pull* in correcting muscle disease symptoms, MyoAAV4A-dual-*Pull* was superior overall in therapeutic outcomes.

While promising, there were several limitations to this study that need to be assessed to more fully evaluate the efficacy of this approach. One factor is that the relatively brief six-week study limited the ability to evaluate or confirm long-term therapeutic durability. Also, the use of only male mice prevented the evaluation of sex as a biological variable which has been shown to be a factor in some AAV-mediated gene therapies.^9^ Studies that are male/female balanced and that incorporate longer time points and aged (more advanced disease progression) mice in future evaluations will be necessary for further validation of the MyoAAV4A-dual-*Pull* approach. Another important factor to consider for this and other disorders where liver fragility may be a concern, is the timing of treatment in human studies. For GSD IIIa, liver involvement (hepatomegaly, hypoglycemia, hyperlipidemia, and elevated hepatic transaminases)^10^ is one of the earlier manifestations of disease while progressive liver fibrosis, hepatic failure, and other complications can occur later.^10^ Thus, attention must be paid to individual patient disease stages, particularly with regards to liver fragility.

The consistent results observed across multiple muscles comprised of a various fiber types strengthen the conclusion that MyoAAV4A is an advancement over AAV9 when muscle is a therapeutic target. While both of the MyoAAV capsids achieved ∼95% glycogen clearance in skeletal and cardiac muscle tissues compared to untreated controls, AAV9 resulted in only ∼50% correction.^2^ Overall, the most compelling success of this story was the high therapeutic efficacy achieved with MyoAAV4A at only one-tenth the dose of AAV9 while maintaining impressive functional recovery in skeletal muscle. Furthermore, the study’s humanized mice data confirmed the ability of MyoAAV4A-dual-*Pull* to sufficiently transduce human hepatocytes.^2^ While incorporating the use of engineered capsids that favor the enhanced muscle tropism may have so successfully detargeted the liver that no therapeutic effect would be possible, this was found not to be the case.

These findings support consideration of MyoAAV4A as a preferred candidate for future clinical studies. Liao et al. provide compelling evidence that the use of myotropic engineered capsids can overcome the barrier of systemic gene delivery for GSD-IIIa by providing effective muscle transduction while maintaining therapeutic effects in the liver at a significantly reduced vector dosage. In sum, MyoAAV4A provided the push that the dual-*Pull* vector needed to advance GSD-IIIa gene therapy closer toward a viable treatment for human patients.

## Declaration of interests

The authors declare no competing interests.

## References

[1] Chand D., Mohr F., McMillan H., Tukov F.F., Montgomery K., Kleyn A., Sun R., Tauscher-Wisniewski S., Kaufmann P., Kullak-Ublick G. (2021). Hepatotoxicity following administration of onasemnogene abeparvovec (AVXS-101) for the treatment of spinal muscular atrophy. J. Hepatol..

[2] Liao K.A., Han S.O., Barzi M., Yi H., Eisner W., Bissig-Choisat B., Bissig K.D., Sun B. (2025). High-potency MyoAAV capsids enhanced skeletal muscle correction in a mouse model of GSD IIIa. Mol. Ther. Methods Clin. Dev..

[3] Liu J., Koay T.W., Maiakovska O., Zayas M., Grimm D. (2023). Progress in Bioengineering of Myotropic Adeno-Associated Viral Gene Therapy Vectors. Hum. Gene Ther..

[4] Weinmann J., Weis S., Sippel J., Tulalamba W., Remes A., El Andari J., Herrmann A.K., Pham Q.H., Borowski C., Hille S. (2020). Identification of a myotropic AAV by massively parallel in vivo evaluation of barcoded capsid variants. Nat. Commun..

[5] Tabebordbar M., Lagerborg K.A., Stanton A., King E.M., Ye S., Tellez L., Krunnfusz A., Tavakoli S., Widrick J.J., Messemer K.A. (2021). Directed evolution of a family of AAV capsid variants enabling potent muscle-directed gene delivery across species. Cell.

[6] Lim J.A., Choi S.J., Gao F., Kishnani P.S., Sun B. (2020). A Novel Gene Therapy Approach for GSD III Using an AAV Vector Encoding a Bacterial Glycogen Debranching Enzyme. Mol. Ther. Methods Clin. Dev..

[7] Lim J.A., Kishnani P.S., Sun B. (2022). Suppression of pullulanase-induced cytotoxic T cell response with a dual promoter in GSD IIIa mice. JCI Insight.

[8] Hinderer C., Katz N., Buza E.L., Dyer C., Goode T., Bell P., Richman L.K., Wilson J.M. (2018). Severe Toxicity in Nonhuman Primates and Piglets Following High-Dose Intravenous Administration of an Adeno-Associated Virus Vector Expressing Human SMN. Hum. Gene Ther..

[9] Abdelhamid L., Mazor R. (2025). Can Sex-based Variations in the Immune Responses to AAV Gene Therapy Affect Safety and Efficacy? A Review of Current Understanding. AAPS J..

[10] Schreuder A.B., Rossi A., Grunert S.C., Derks T.G.J., Adam M.P., Bick S., Mirzaa G.M. (1993). GeneReviews((R)).

